# POU4F1 promotes the resistance of melanoma to BRAF inhibitors through MEK/ERK pathway activation and MITF up-regulation

**DOI:** 10.1038/s41419-020-2662-2

**Published:** 2020-06-12

**Authors:** Lin Liu, Qiao Yue, Jingjing Ma, Yu Liu, Tao Zhao, Weinan Guo, Guannan Zhu, Sen Guo, Shiyu Wang, Tianwen Gao, Chunying Li, Qiong Shi

**Affiliations:** Department of Dermatology, Xijing Hospital, Fourth Military Medical University, 710032 Xi’an, Shaanxi China

**Keywords:** Cancer therapeutic resistance, Targeted therapies, Melanoma

## Abstract

BRAF inhibitors (BRAFi) have shown remarkable clinical efficacy in the treatment of melanoma with BRAF mutation. Nevertheless, most patients end up with the development of BRAFi resistance, which strongly limits the clinical application of these agents. POU4F1 is a stem cell-associated transcriptional factor that is highly expressed in melanoma cells and contributes to BRAF-activated malignant transformation. However, whether POU4F1 contributes to the resistance of melanoma to BRAFi remains poorly understood. Here, we report that over-expressed POU4F1 contributed to the acquired resistance of melanoma cells to Vemurafenib. Furthermore, POU4F1 promoted the activation of ERK signaling pathway via transcriptional regulation on MEK expression. In addition, POU4F1 could increase the expression of MITF to retain the resistance of melanoma cells to BRAFi. Collectively, our findings reveal that POU4F1 re-activates the MAPK pathway by transcriptional regulation on MEK expression and promotes MITF expression, which ultimately results in the resistance to BRAFi in melanoma. Our study supports that POU4F1 is a potential combined therapeutic target with BRAFi therapy for melanoma.

## Introduction

The incidence of melanoma has always been increasing worldwide and the median survival time of patients with advanced melanoma is only 6–8 months^1^. In the last 10 years, the discovery of high mutation rate of B-Raf Proto-Oncogene (BRAF) in melanoma provides the patients with the choice of drugs targeting BRAF that is involved in mitogen-activated protein kinase (MAPK) pathway, which has significantly improved the patients’ survival^2,3^. However, melanoma could relapse and progress quickly in about six months from the start of BRAFi monotherapy, which greatly hampers the clinical application of BRAFi^4^. Recent studies have indicated that the resistance of melanoma to BRAFi is closely related to the reactivation of MAPK pathway caused either by the mutation or the overexpression of the molecules in MAPK pathway or by the aberrant crosstalk between MAPK and other pathways like phosphatidylinositol 3-kinase (PI3K) pathway^5,6^.

Since melanoma derives from melanocytes that originate from precursor cells in the neural crest^7,8^, melanoma cells share some stem cell-like phenotypes with neural crest cells and express similar transcription factors that lead to those phenotypes^8^. The roles of these transcription factors, especially microphthalmia-associated transcription factor (MITF) and SRY (sex determining region Y)-Box (SOX) family^9^, in the resistance of melanoma to targeted treatments have been investigated by some previous studies. In melanoma cells resistant to MAPK inhibitors including BRAFi, a state of low expressions of MITF and SOX10 could induce high levels of tyrosine kinase receptors, such as AXL receptor tyrosine kinase (AXL) and epidermal growth factor receptor (EGFR) that help to maintain the resistance^10,11^. However, the overexpression of MITF has also been demonstrated to drive resistance, indicating a complex function of MITF in the resistance of melanoma to targeted therapy^12^. In addition, SOX2 that is a stem cell marker essential for the formation of pluripotent cells in the embryo^13^ is found to be up-regulated and activate signal transducer and activator of transcription 3 (STAT3) in melanoma cells resistant to BRAF-targeted therapy^14^. These studies emphasize the importance of stem cell-associated transcription factors to BRAFi resistance.

POU Class 4 Homeobox 1 (POU4F1), a member of the POU domain family transcription factors, is also a stem cell-associated transcription factor expressed in proliferating precursor cells in the neural crest^15,16^. POU4F1 regulates the apoptosis of neuronal cells as evidenced by the fact that the mice with POU4F1 deficiency display the apoptosis of certain sensory neurons^17^, which is due to the transcriptional inactivation of anti-apoptotic Bcl-2 and Bcl-xL^18,19^. Furthermore, POU4F1 modulates the activity of p53 in a protein–protein interaction manner^20^. The function of POU4F1 in the development of melanocytic lineage has not been determined^21^, but the oncogenic role of POU4F1 in melanoma has been observed recently. The inhibition of POU4F1 in melanoma cells causes DNA double-strand breaks and subsequent activation of p53, which inhibits cell senescence under the synergetic effect of BRAF activation and induces malignant transformation of melanocytes^22^. Therefore, POU4F1 may be involved in the resistance of melanoma to BRAFi. Based on these previous findings, the current preclinical study was undertaken to explore the role of POU4F1 in the formation of BRAFi resistance in melanoma as well as the molecular mechanism involved in the process.

## Materials and methods

### Clinical samples and cell lines

Normal human melanocytes (NHMs) were isolated and cultured as described previously^23^. Melanocyte cell lines and melanoma cell lines were purchased from American Tissue Culture Collection (ATCC) and cultured as recommended by the supplier. Tissue sections for immunohistochemistry staining were taken from 20 melanoma patients (10 patients at primary stage and 10 patients at metastatic stage) and 6 patients with nevus after histological confirmation. All the patients were recruited from Xijing Hospital (Xi’an, China) between September 2016 and July 2019. All subjects were Chinese Han people with no previous systemic treatment. Informed consent was obtained from all the patients. The research protocol was designed and executed according to the principles of the Declaration of Helsinki and was approved by the ethics review board of Fourth Military Medical University.

### Antibodies and reagents

The antibodies against POU4F1 (ab81213) and MITF (ab12039) were purchased from Abcam (Cambridge, MA, USA). The antibodies against POU4F1 for ChIP assay (sc-8429 ×) was purchased from Santa Cruz Biotechnology (Santa Cruz, CA., USA). The antibodies against ERK (#4696), p-ERK (Thr202/Tyr204, #4370), MEK (#4694), p-MEK (Ser217/221, #3985), Actin (#3700) and Ki-67 (#9449) were purchased from Cell Signaling Technology, Inc. (Danvers, MA, USA). Vemurafenib (#918504-65-1) and PD98059 (#167869-21-8) were purchased from MedChemExpress (New Jersey, USA).

### Quantitative RT-PCR

Total RNA was extracted using TRIzol reagent (Invitrogen) and then reversely transcribed into single-strand cDNA using reverse transcription reagents (TaKaRa, Dalian, China) according to the manufacturer’s instructions. Quantitative RT-PCR (qRT-PCR) was carried out using the SYBR Mix (TaKaRa) and Bio-Rad Multicolor Real-time PCR Detection System (iQTM5, Bio-Rad). And the cycling conditions were set as 95 °C for 2 min followed by 40 cycles of denaturation at 95 °C for 5 s, annealing at 55 °C for 10 s and extension at 72 °C for 15 s. The relative abundance of mRNA expression of a control sample was arbitrarily designated as 1. The primer sequences used are as follow: forward, 5′ - ACGCACGAACTGAGTCGAAA - 3′, reverse, 5′ - CACTTCCCGGGATTGGAGAG - 3′ for POU4F1; 5′ - GGGCTTCTATGGTGCGTTCTA - 3′, reverse, 5′- CCCACGGGAGTTGACTAGGAT - 3′ for MEK; forward, 5′ - CTCACAGCGTGTATTTTTCCCA - 3′, reverse, 5′ - ACTTTCGGATATAGTCCACGGAT - 3′ for MITF.

### Western blotting

Proteins were extracted from cells and quantified using BCA protein assay kit (Thermo Scientific-Pierce, Rockford, Illinois, USA). Equal amounts (20–25 μg) of protein were separated by 10% SDS-PAGE polyacrylamide gels (Bio-Rad) and then transferred to PVDF membranes (Millipore, Nottingham, UK). After blocking in a solution of 5% nonfat dry milk diluted in Tris-buffered saline for 1 h, the blots were incubated with primary antibodies and then with horseradish peroxidase-conjugated secondary antibodies. Bound antibodies were detected using the enhanced chemiluminescence (Pierce) and the ECL Western blotting detection system (Millipore).

### Immunohistochemistry

Dewaxed sections (4 μm) were hydrated with heated 1x TE buffer and incubated in goat serum to block nonspecific binding. Then, the sections were incubated overnight at 4 °C in anti-human primary antibodies for POU4F1 and Ki-67 diluted in 1x TBS. The Dako REALTM EnVisionTM Detection System (Dako, Denmark, A/S) was used to detect primary antibodies according to the manufacturer’s instructions. Sections were then incubated in Fast Red solution, counterstained with hematoxylin and mounted with glycerol. Controls included sections with TBS only and nonrelevant isotype mouse monoclonal antibody. Immunohistochemistry was interpreted by a pathologist blinded to the other data.

The evaluation of staining scores was described previously^24^. Briefly, the percentages of staining-positive cells were scored into four categories: 0 (0%), 1 (1–33%), 2 (34–66%) and 3 (67–100%). The staining intensities were scored into four grades: 0 (none), 1 (week), 2 (moderate) and 3 (strong). The final staining score was defined as the product of the percentage and intensity scores.

### Small interference RNA and transfection

The siRNAs targeting on MITF were purchased from GenePharma (Shanghai, China). The sequences were as follows: sense, 5′ - AGCAGUACCUUUCUACCACTT - 3′, antisense, 5′ - GUGGUAGAAAGGUACUGCUTT - 3′. Transfection of siRNAs was carried out in Opti-MEM medium (Invitrogen) using Lipofectamine 3000 reagent (Invitrogen) according to the manufacturer’s transfection protocol.

### Plasmid vectors and transfection

The POU4F1 overexpression plasmid (RC216284) and the according control plasmid pCMV6-Entry ((PS100001) were purchased from ORIGENE. The POU4F1 shRNA plasmid (sc-29839-SH) was purchased from Santa Cruz biotechnology. Transfection of plasmid vectors was carried out in Opti-MEM medium (Invitrogen) using Lipofectamine 3000 reagent (Invitrogen) according to the manufacturer’s transfection protocol.

### Cell viability

Cells were seeded at 5000 per well onto flat-bottom 96-well plates and allowed to grow for 24 h followed by indicated treatments. CCK-8 reduction assay kits (Beyotime Biotechnology, Shanghai, China) were used as a quantitative index of cell viability according to the manufacturer’s instructions. The absorbance was measured at 450 nm in a Microplate Reader (Model 680, Bio-Rad).

### Colony formation assay

Cells that grew for 24 h followed by transfection were seeded at 2000 per well onto six-well culture plates and treated as indicated. These cells were allowed to grow for another 12 days under constant treatment. Cells were then fixed with methanol and stained with crystal violet (Sigma-Aldrich). Colonies were counted with Image J software.

### ChIP assay

ChIP experiments were performed according to the manufacturer’s recommended procedures of SimpleChIP® Plus Sonication Chromatin IP Kit (#56383, Cell Signaling Technology). Briefly, cells were fixed with formaldehyde and lysed. The chromatin was fragmented by ultrasonic treatment to obtain 200–1000 bp chromatin fragments. The recovered supernatant fraction was incubated with antibodies overnight on a rotor at 4 °C. After washing, the precipitated POU4F1 protein-DNA complexes were recovered by using protein Protein G Magnetic Beads at 4 °C for 16 h. DNA was then purified with phenol/chloroform. A fraction was used as the PCR template to detect the presence of promoter sequences of MEK and MITF. The enrichment (100% input) was analyzed by real time PCR calculated using 100% × 2^(adjusted Input − Ct(IP))^, where adjusted Input = Ct(Input) − 5.644. The primer sequences used are as follow: MEK: forward, 5′ - ACCAGGGTTGGAGAAGACTAC - 3′, reverse, 5′ - GGGGCTAGGTCTACCGGG - 3′; MITF: forward, 5′ - GCCCAGTTGTCACTGTAGCC - 3′, reverse, 5′ - AACTCTGCTCAGTGGTCATTCT - 3′.

### Statistics

Analysis was carried out using either GraphPad Prism version 5 (La Jolla, California, USA) or SPSS version 16 (SPSS Inc., Chicago, Illinois, USA). Unless otherwise noted, the results were analyzed by two-tailed Student’s *t* test, and the results are presented as mean ± SEM through at least 3 independent experiments. A *P*-value less than 0.05 was considered significant.

## Results

### POU4F1 is highly expressed in melanoma

**Fig. 1:**
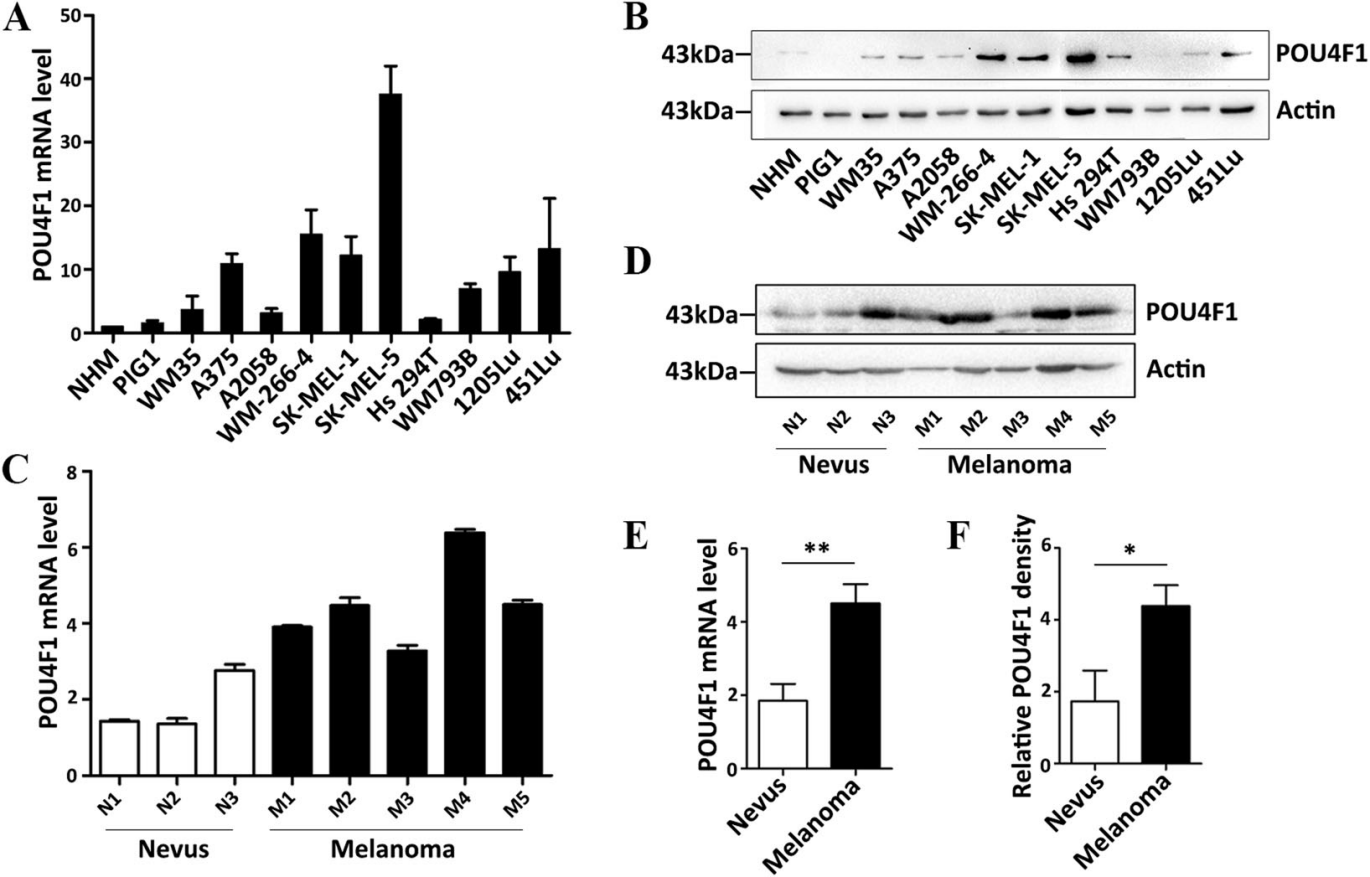
POU4F1 was highly expressed in melanoma. **a** POU4F1 mRNA level was analyzed by qRT-PCR in NHMs, melanocyte cell line (PIG1) and melanoma cell lines, *n* = 3. **b** POU4F1 protein expression was analyzed by immunoblotting in NHMs, melanocyte cell line and melanoma cell lines. **c** POU4F1 mRNA level was analyzed by qRT-PCR in nevus (N) and melanoma (M) tissues, *n* = 3. **d** Statistics analysis on the mRNA expression level of POU4F1 in nevus and melanoma tissues. **e** POU4F1 protein expression was analyzed by immunoblotting in nevus (N) and melanoma (M) tissues. **f** Densitometry analysis on the protein level of POU4F1 in nevus and melanoma tissues. Data are mean ± SEM, *p < 0.05, **p < 0.01.

POU4F1 expression was analyzed in human melanoma cell lines and non-malignant melanocytes. Most melanoma cell lines (7/10, 70%) expressed high mRNA level of POU4F1 compared with NHMs or PIG1 cells that are an immortalized melanocytic cell line (Fig. 1a). Consistently, POU4F1 was increased in most melanoma cell lines at protein level (Fig. 1b). High expression of POU4F1 was also observed at both mRNA level (Fig. 1c, e) and protein level (Fig. 1d, f) in melanoma tissues compared with nevus tissues. These results demonstrate the overexpression of POU4F1 in melanoma, indicating a potential function of POU4F1 in melanoma progression.

### The expression level of POU4F1 is correlated with the progression of melanoma

**Fig. 2:**
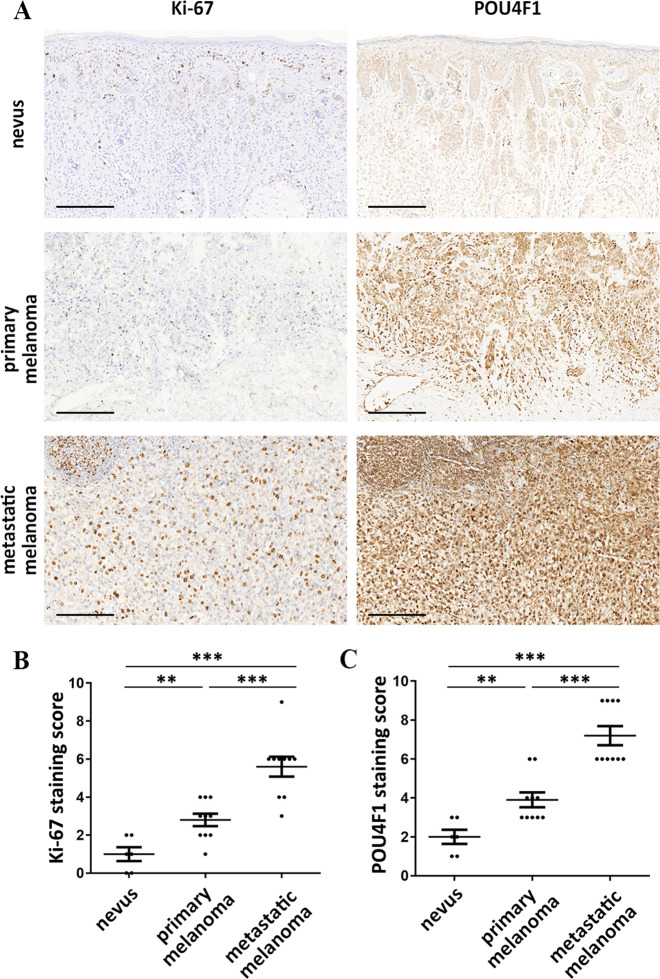
The expression level of POU4F1 was associated with Ki-67 level and the progression of melanoma. **a** Representative immunohistochemistry staining images of Ki-67 and POU4F1 expressions in nevus and melanoma tissues, bar = 200 μm. **b** Quantitative analysis of Ki-67 staining score. **c** Quantitative analysis of POU4F1 staining score. (nevus *n* = 6, primary melanoma *n* = 10, metastatic melanoma *n* = 10) Data are mean ± SEM, **p < 0.01, ***p < 0.001.

To further confirm the oncogenic role of POU4F1 in melanoma, we analyzed the expression levels of POU4F1 and Ki-67 in nevus tissues and melanoma tissues from different progression stages. The expression of human Ki-67 protein is strictly associated with cell proliferation and is absent from resting cells^25^. The fraction of Ki-67-positive tumor cells is often correlated with clinical stages, indicating Ki-67 as an important and precise indicator for the progression and prognosis of tumors including melanoma^26^. We performed immunohistochemistry to evaluate both POU4F1 and Ki-67 expression scores in tissue specimens from 20 melanoma cases as well as 6 noncancerous nevus cases (Fig. 2a). As expected, Ki-67 expression scores increased in primary melanoma tissues compared with nevus tissues, and further elevated in metastatic melanoma tissues (Fig. 2b). Moreover, a similar increasing tendency of POU4F1 expression scores was observed in our samples (Fig. 2c). This parallel change of Ki-67 and POU4F1 expressions demonstrates that POU4F1 is a potential biomarker for determining the status of melanoma patients.

### POU4F1 is involved in the resistance of melanoma cells to BRAFi

**Fig. 3:**
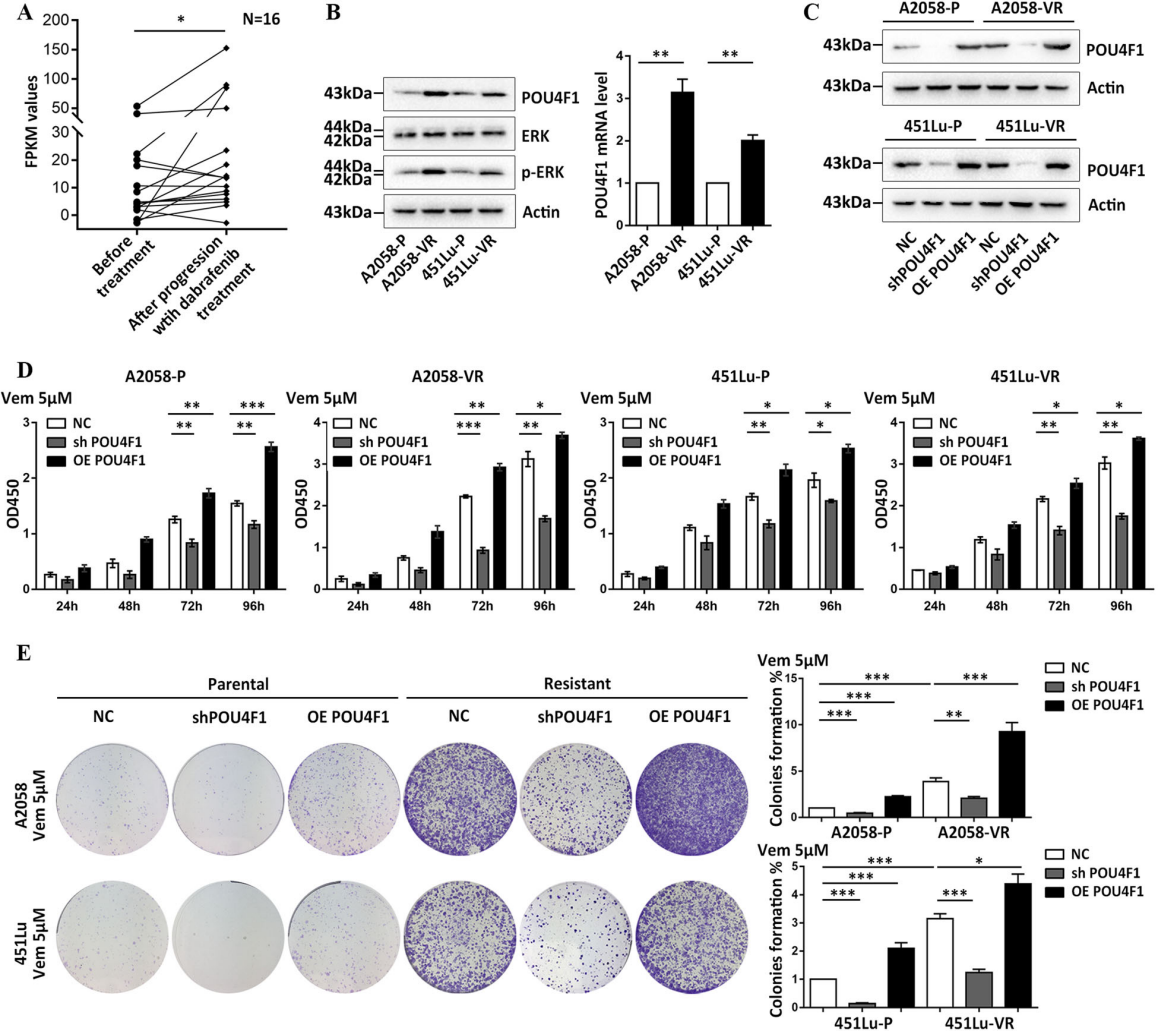
POU4F1 promoted the resistance of melanoma cells to Vemurafenib. **a** The expression level (FPKM values) of POU4F1 in the patients before treatment and after progression with the treatment of dabrafenib. Data were collected from GEO database (GSE50509), *n* = 16. **b** The protein level of POU4F1, ERK and phosphorylated ERK and the mRNA level of POU4F1 were analyzed by immunoblotting and qRT-PCR in parental and resistant cells. **c**. The knockdown and overexpressing efficiency of POU4F1 shRNA and plasmid in parental and resistant cells of A2058 and 451Lu. **d** Cell viability was evaluated by CCK8 assay in both parental and resistant cells under the treatment of Vemurafenib, *n* = 3. **e** Cell colony formation of parental and resistant cells was measured by colony formation assay under the treatment of Vemurafenib, *n* = 3. Data are presented as the mean ± SEM, *p < 0.05, **p < 0.01, ***p < 0.001. NC negative control. sh short hairpin RNA. OE over expressing. P parental cells. VR Vemurafenib-resistant cells. Vem Vemurafenib.

In order to explore the potential role of POU4F1 in the resistance of melanoma to BRAFi, we first performed POU4F1 expression analysis using data from the GEO database. The dataset of GSE50509 included the mRNA profile data in melanoma tissue samples from the patients before the treatment with dabrafenib that is a BRAFi and at the time of tumor progression. The data showed that POU4F1 expression in melanoma tissues increased significantly after the occurrence of resistance to dabrafenib (*P* = 0.0357, Fig. 3a).

To test the function of POU4F1 in BRAFi resistance, we established two melanoma cell lines (A2058 and 451Lu) with acquired resistance to Vemurafenib (Fig. S1). We assessed the expression level of POU4F1 in both parental cells and resistant cells (validated by the activation of ERK) and observed a significant increase of POU4F1 expression in resistant cells (Fig. 3b). We regulated the expression of POU4F1 by using overexpression plasmids and POUF41 shRNAs (overexpressing and interfering efficiencies shown in Fig. 3c) and found that the overexpression of POU4F1 promoted the resistance to Vemurafenib in both parental and resistant cells, while the down-regulation of POU4F1 sensitized melanoma cells to Vemurafenib (Fig. 3d). Further colony formation assay showed a significantly increased number of colonies in POU4F1-overexpressed cells and a down-regulated number of colonies in cells with POU4F1 knockdown in parental and resistant cells (Fig. 3e). These results indicate that POU4F1 promotes the activation of ERK and facilitates the resistance of melanoma cells to BRAFi.

### POU4F1 activates the MEK/ERK pathway to render melanoma resistant to BRAFi

**Fig. 4:**
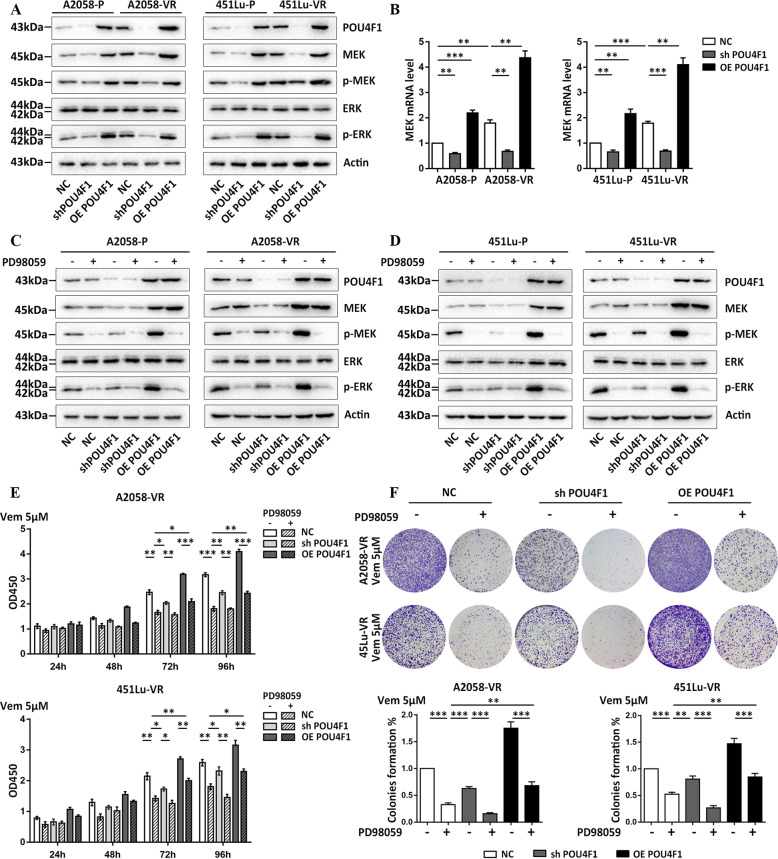
POU4F1 activated MEK/ERK pathway to retain the resistance towards Vemurafenib in melanoma. **a** The expressions of MEK, p-MEK, ERK and p-ERK were analyzed by immunoblotting in parental and resistant melanoma cells with indicated treatments. **b** The mRNA expression level of MEK was detected by qRT-PCR in parental and resistant melanoma cells with POU4F1 knockdown or overexpression, *n* = 3. **c**, **d** The expressions of MEK, p-MEK, ERK and p-ERK were analyzed by immunoblotting in parental and resistant melanoma cells under the treatment of MEK inhibitor (PD98059). **e** Cell viability of resistant cells was measured by CCK8 assay with indicated treatment. *n* = 3. **f** Cell colony formation of resistant cells was measured by colony formation assay with indicated treatment, *n* = 3. Data are presented as the mean ± SEM, *p < 0.05, **p < 0.01, ***p < 0.001. NC negative control. sh short hairpin RNA. OE over expressing. P parental cells. VR Vemurafenib-resistant cells. Vem Vemurafenib.

We went on to explore the mechanism underlying POUF41-induced activation of ERK and resistance of melanoma to BRAFi in upstream MAPK pathway. Our immunoblot analysis showed that the overexpression of POU4F1 in both parental and resistant cells elevated the protein levels of MEK and p-MEK and p-ERK, whereas the knockdown of POU4F1 led to opposite effect (Fig. 4a). Consistently, the qRT-PCR assay observed increased mRNA level of MEK in POU4F1-overexpressed cells and decreased mRNA level of MEK in POU4F1-knockdown cells (Fig. 4b). To further validate the participation of MEK/ERK pathway in POU4F1-induced cell resistance to BRAFi, we treated cells with PD98059^27^, a specific inhibitor of MEK, in combination with the interference or the overexpression of POU4F1. As expected, PD98059 significantly suppressed the phosphorylation of MEK and ERK without influencing the expressions of MEK and ERK (Fig. 4c, d). Notably, the inhibition on MEK/ERK pathway was more obvious in the cells treated with PD98059 compared with the cells transfected with POU4F1 shRNA, implicating additional mechanism underlying the activation of MAPK pathway that is independent on POU4F1 in the process. In addition, our CCK8 (Fig. 4e) and colony formation assays (Fig. 4f) showed that blocking either the expression of POU4F1 by shRNA or the activation of MEK/ERK pathway by PD98059 restored the sensitivity of melanoma cells to Vemurafenib. Moreover, PD98059 suppressed the effect of POU4F1 overexpression on the resistance of melanoma cells to Vemurafenib. These results demonstrate that POU4F1 promotes the resistance of melanoma cells to BRAFi via the activation of MEK/ERK pathway. However, both the CCK8 and colony formation assays showed significantly higher cell growth in POU4F1-overexpressed melanoma cells under the treatment of PD98059 (Fig. 4e, f), indicating that MEK/ERK is not the only downstream effecter of POU4F1 in the formation of BRAFi resistance in melanoma.

### MITF contributes to the resistance of melanoma to BRAFi induced by POU4F1

**Fig. 5:**
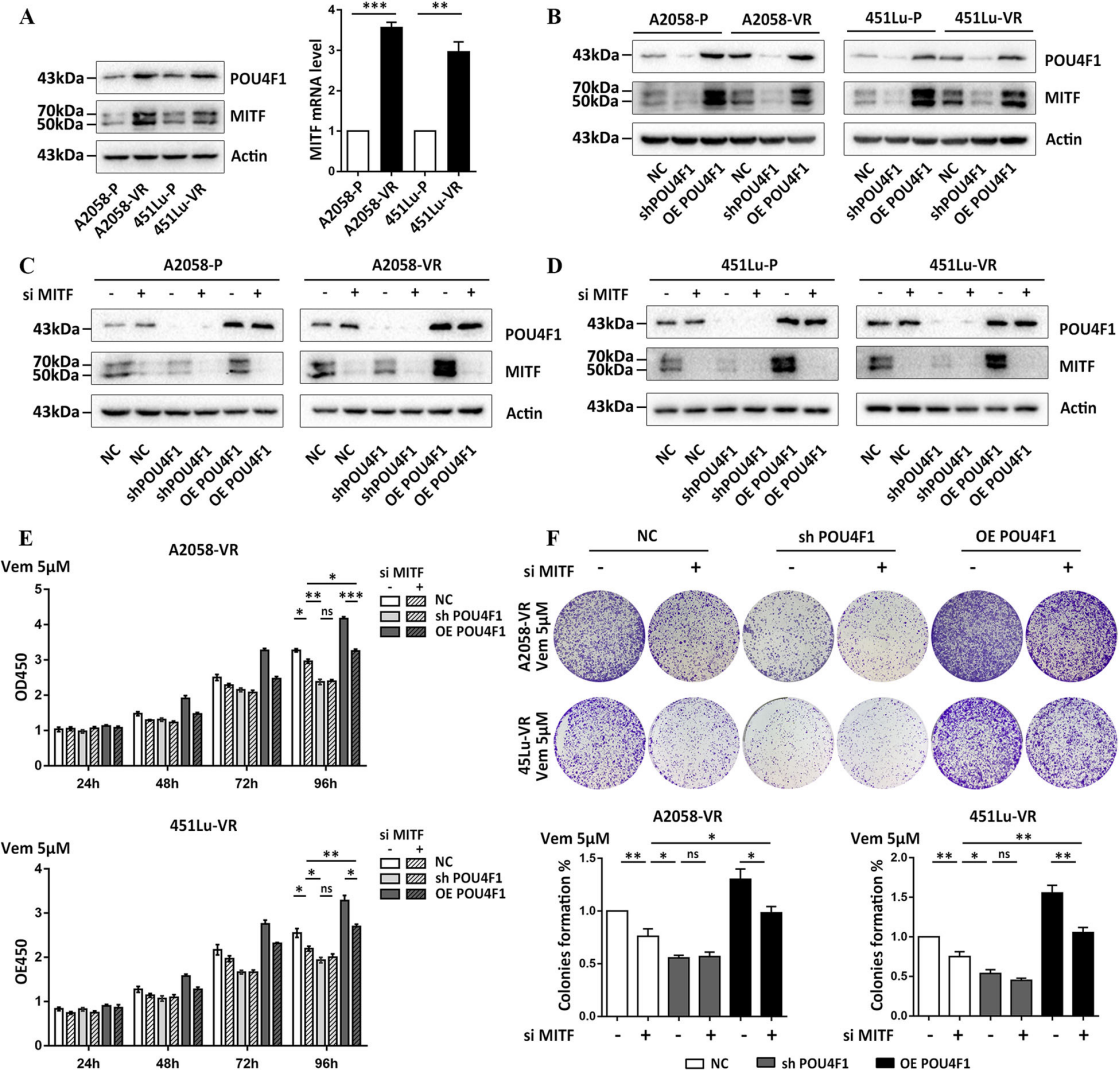
POU4F1 contributed to melanoma resistance to Vemurafenib by promoting the expression of MITF. **a** The protein and mRNA expression levels of MITF were analyzed in parental and resistant melanoma cells, *n* = 3. **b** The expression of MITF was analyzed by immunoblot in parental and resistant melanoma cells with indicated treatments. **c**, **d** The expressions of POU4F1 and MITF were analyzed in A2058 and 451Lu cells transfected with MITF siRNA in combination with POU4F1 knockdown or overexpression. **e** Cell viability was estimated by CCK8 assay in resistant cells with indicated treatment, *n* = 3. **f** The sensitivity of resistant cells to Vem were evaluated by colony formation assay with indicated treatment, *n* = 3. Data are presented as the mean ± SEM, ns= no significant, *p < 0.05, **p < 0.01, ***p < 0.001. NC negative control. sh short hairpin RNA. OE over expressing. P parental cells. VR Vemurafenib-resistant cells. Vem Vemurafenib.

MITF, a master regulator of melanocyte survival and function, is involved in the formation of BRAFi resistance via activating peroxisome proliferator-activated receptor-γ co-activator 1α (PGC1α) and mitochondrial activity^28,29^. We found that POU4F1 and MITF were parallelly increased in Vemurafenib-resistant melanoma cells compared with parental cells (Fig. 5a). Subsequent immunoblot assay showed that the expression of MITF was increased in melanoma cells with POU4F1 overexpression and decreased in melanoma cells with POU4F1 knockdown (Fig. 5b), proving that the expression of MITF was regulated by POU4F1. We therefore down-regulated the expression of MITF in both parental and resistant melanoma cells by using MITF siRNA (interfering efficiencies shown in Fig. 5c, d) and found that knocking down MITF also restored the sensitivity of melanoma cells to Vemurafenib (Fig. 5e, f), However, this effect was significantly weaker than knocking down POU4F1, demonstrating that MITF is one of the downstream targets of POU4F1 in the formation of resistance of melanoma to BRAFi.

### POU4F1 directly binds to the promoters of MEK and MITF to promote their expressions

**Fig. 6:**
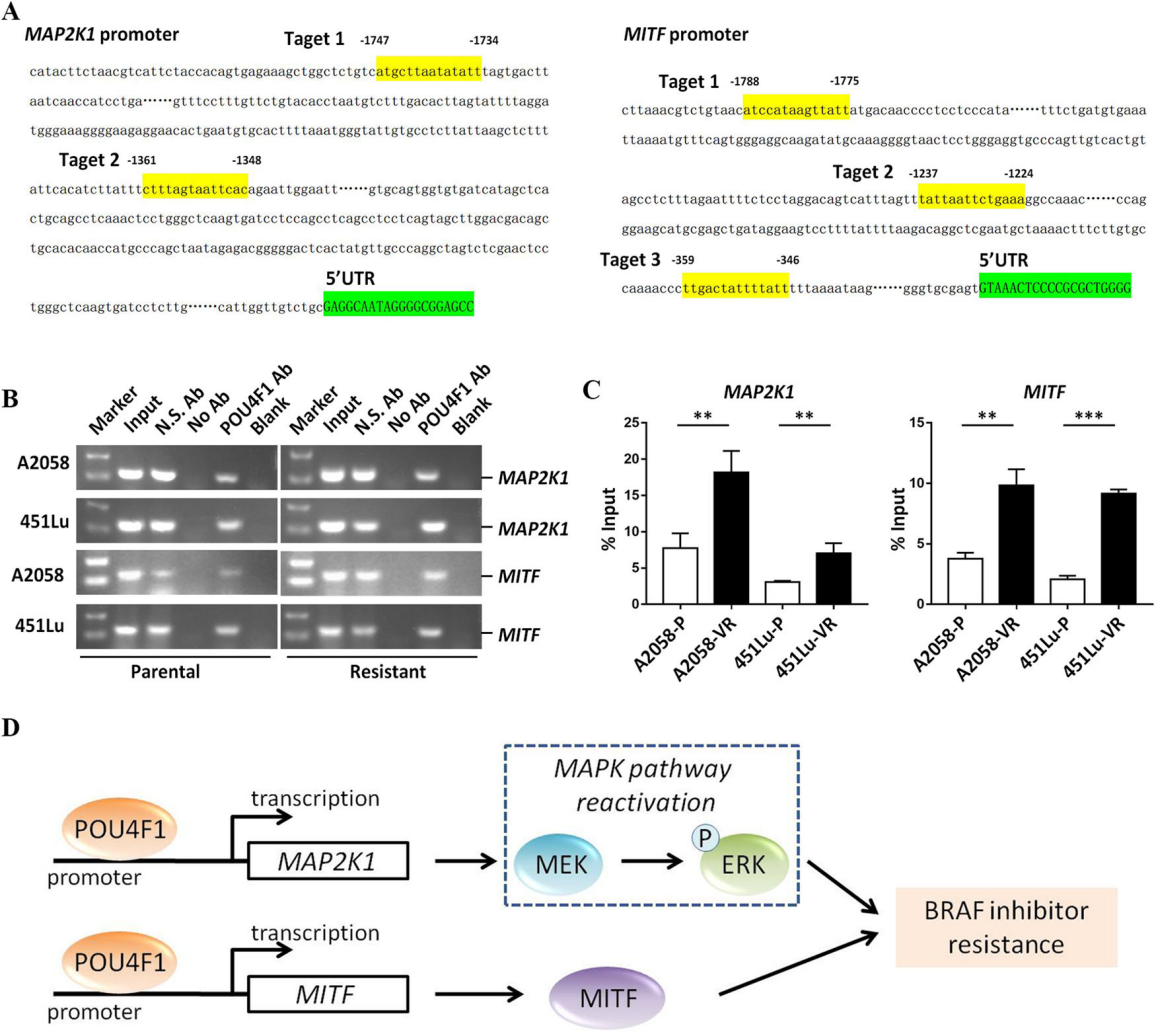
POU4F1 bound to the promoter of *MAP2K1* and *MITF*. **a** Predicted binding targets marked on the sequence of *MAP2K1* (left) and *MITF* (right) promoter. The targets were predicted by JASPAR website. **b** ChIP assay was performed to analyze the direct binding of POU4F1 on the promoter of *MAP2K1* and *MITF*. Lane 1, DNA ladder (Marker). Lane 2, input chromatin prior to immunoprecipitation (Input). Lane 3, immunoprecipitation with a nonspecific antibody (N.S. Ab). Lane 4, immunoprecipitation without antibody (No Ab).Lane 5, immunoprecipitation with the POU4F1 antibody (POU4F1 Ab). Lane 6, PCR production without chromatin (Blank). **c** Quantitative analysis of the ChIP assay, *n* = 3. **d** The proposed model of the role of POU4F1 in the resistance of melanoma to BRAFi. Overexpressed POU4F1 transcriptionally promotes the expression of MEK (*MAP2K1*) and MITF in transcriptional manners, reactives MAPK pathway and finally leads to the resistant phenotype of melanoma cells to BRAFi. Data are presented as the mean ± SEM, **p < 0.01, ***p < 0.001. P parental cells. VR Vemurafenib-resistant cells.

Given that POU4F1 is a transcriptional factor, we predicted the binding sites of POU4F1 on the promoter regions of *MAP2K1* (gene of MEK) and *MITF* in JASPAR database and found two potential binding sites of POU4F1 on *MAP2K1* promoter and three ones on *MITF* promoter, respectively (Fig. 6a). Subsequent CHIP assays detected amplified DNA bands using specific primers designed according to predicted POU4F1 binding sites within *MAP2K1* and *MITF* promoters in the group of chromatin immunoprecipitated with POU4F1 antibody (Fig. 6b), which was more significant in the cells resistant to Vemurafenib compared with parental cells (Fig. 6c). These results verify that POU4F1 transcriptionally promotes the expressions of MEK and MITF in melanoma cells.

## Discussion

Our study provides the evidence for the contribution of POU4F1 to the resistance of melanoma cells to BRAFi via activating MEK/ERK pathway and MITF. Initially, POU4F1 directly binds to the promoter regions of the gene of MEK and MITF and transcriptionally promotes their expressions. Elevated MEK further induces the phosphorylation of ERK that is a key kinase in MAPK pathway. Finally, the activation of both MEK/ERK pathway and MITF mediates the formation of the resistance to BRAFi in melanoma cells (Fig. 6d).

The resistance towards BRAFi is very common in clinical practice of the therapy for malignancies. The re-activation of MAPK pathway is of particular importance to the resistance to BRAFi therapy^5^. Previous studies have demonstrated several mechanisms underlying the re-activation of MAPK path way in BRAFi-treated cells, including the activation of receptor tyrosine kinases (RTKs), secondary mutations of genes involved in MAPK pathway including BRAF, NRAS, KRAS and MEK and the crosstalk with other pathways like PI3K/Akt^6,30,31^. Supplementary to these previous findings, our study found that elevated POU4F1 could activate MEK/ERK that is a key link in the whole MAPK pathway, thus leading to the resistance to BRAFi in melanoma cells, which is a novel mechanism for MAPK pathway reactivation in melanoma under BRAFi treatment.

The combined therapy of BRAF and MEK inhibitors has been proved to improve the rate of progression-free survival in melanoma patients compared with BRAFi alone^32,33^. However, since MEK regulates key cellular processes in almost all cells that require frequent proliferation^34,35^, MEK inhibitors could cause serious adverse reactions such as severe skin manifestations, diarrhea and fatigue, which often requires dose reduction or even drug withdrawal^35,36^. POU4F1 is mainly expressed in nervous system during embryogenesis and its expression is terminated in the majority of organs in adults^15,37^. A previous study has described that POU4F1 could only be detected in melanoma cell lines rather than cultured melanocytes^22^, and our study demonstrates a similar result not only in cell lines but also in nevus and melanoma tissues. In this aspect, POU4F1 could be a better target for combined therapy with BRAFi than MEK.

A growing body of evidence identifies MITF as a dichotomous molecule involved in the resistance to MAPK inhibition therapy^38^. BRAFi induces MITF depletion and thus activate RTKs, such as EGFR and AXL, that lead to a resistant phenotype^10,11^. However, the treatment of BRAF/MEK inhibitors could also up-regulated the expression of MITF^12,39^, which subsequently activates antiapoptotic BCL2A1^40^ and PGC1α that is a master regulator of mitochondrial biogenesis and oxidative phosphorylation^28^, thus leading to the formation of resistance to MAPK pathway inhibitors^41^. Our study showed that POU4F1 directly bound to the promoter region of MITF and transcriptionally promoted the expression of MITF, which is a novel upstream mechanism for MITF activation in BRAFi resistance. It has to be noted that some other transcriptional factors are also involved in MITF activation in the formation of BRAFi resistance in melanoma. Paired box 3 (PAX3) is a classic transcriptional inducer of MITF and is necessary for the up-regulation of MITF induced by BRAF/MEK inhibition^42^. Cadherin-associated protein beta 1 (β-catenin) is another known transcription factor that regulates the expression of MITF^43^. Previous studies have found that β-catenin contributes to the formation of BRAFi resistance in melanoma, which, however, is independent on its downstream canonical Wnt-signaling pathway^44,45^. Given that β-catenin could facilitate the proliferation and metastasis of melanoma in an MITF-dependent manner, MITF probably mediates the effect of β-catenin on BRAFi therapy in melanoma patients.

In conclusion, our study demonstrates that POU4F1 is up-regulated in the melanoma cells, which promotes the resistance to BRAFi by the activation of MEK/ERK pathway and MITF. Targeting POU4F1 may be a potential therapeutic approach combined with BRAFi for the therapy of melanoma in the future.

## Supplementary information

supplemental figure legend

supplemental figure S1

## References

[1] Ferlay J (2015). Cancer incidence and mortality worldwide: sources, methods and major patterns in GLOBOCAN 2012. Int. J. Cancer.

[2] Chapman PB (2011). Improved survival with Vemurafenib in melanoma with BRAF V600E mutation. N. Engl. J. Med..

[3] Kim G (2014). FDA approval summary: Vemurafenib for treatment of unresectable or metastatic melanoma with the BRAF V600E mutation. Clin. Cancer Res..

[4] Finn L, Markovic SN, Joseph RW (2012). Therapy for metastatic melanoma: the past, present, and future. BMC Med..

[5] Amaral T (2017). MAPK pathway in melanoma part II - secondary and adaptive resistance mechanisms to BRAF inhibition. Eur. J. Cancer.

[6] Meierjohann S (2017). Crosstalk signaling in targeted melanoma therapy. Cancer Metastasis Rev..

[7] Crane JF, Trainor PA (2006). Neural crest stem and progenitor cells. Annu. Rev. Cell Dev. Biol..

[8] Lang D, Mascarenhas JB, Shea CR (2013). Melanocytes, melanocyte stem cells, and melanoma stem cells. Clin. Dermatol..

[9] Cohen-Solal KA, Kaufman HL, Lasfar A (2018). Transcription factors as critical players in melanoma invasiveness, drug resistance, and opportunities for therapeutic drug development. Pigment Cell Melanoma Res..

[10] Müller J (2014). Low MITF/AXL ratio predicts early resistance to multiple targeted drugs in melanoma. Nat. Commun..

[11] Ji Z (2015). MITF modulates therapeutic resistance through EGFR signaling. J. Invest. Dermatol..

[12] Johannessen CM (2013). A melanocyte lineage program confers resistance to MAP kinase pathway inhibition. Nature.

[13] Mistri TK (2015). Selective influence of Sox2 on POU transcription factor binding in embryonic and neural stem cells. EMBO Rep..

[14] Hüser L (2018). SOX2-mediated upregulation of CD24 promotes adaptive resistance toward targeted therapy in melanoma. Int. J. Cancer.

[15] Latchman DS (1998). The Brn-3a transcription factor. Int. J. Biochem. Cell Biol..

[16] Eng SR, Dykes IM, Lanier J, Fedtsova N, Turner EE (2007). POU-domain factor Brn3a regulates both distinct and common programs of gene expression in the spinal and trigeminal sensory ganglia. Neural Dev..

[17] Huang L (2014). Pou4f1 and pou4f2 are dispensable for the long-term survival of adult retinal ganglion cells in mice. PLoS ONE.

[18] Smith MD, Ensor EA, Coffin RS, Boxer LM, Latchman DS (1998). Bcl-2 transcription from the proximal P2 promoter is activated in neuronal cells by the Brn-3a POU family transcription factor. J. Biol. Chem..

[19] Smith MD (2001). Brn-3a activates the expression of Bcl-xL and promotes neuronalsurvival in vivo as well as in vitro. Mol. Cell. Neurosci..

[20] Hudson CD, Morris PJ, Latchman DS, Budhram-Mahadeo VS (2005). Brn-3a transcription factorblocks p53-mediated activation of proapoptotic target genes Noxa and Bax in vitro and in vivo to determine Cell Fate. J. Biol. Chem..

[21] Besch R, Berking C (2014). POU transcription factors in melanocytes and melanoma. Eur. J. Cell Biol..

[22] Hohenauer T (2013). The neural crest transcription factor Brn3a is expressed in melanoma and required for cell cycle progression and survival. EMBO Mol. Med..

[23] Cario M, Taieb A (1993). Isolation and culture of epidermal melanocytes. Methods Mol. Biol..

[24] Guo S (2016). Serum miR-16: a potential biomarker for predicting melanoma prognosis. J. Invest. Dermatol..

[25] Scholzen T, Gerdes J (2000). The Ki-67 protein: from the known and the unknown. J. Cell. Physiol..

[26] Nielsen PS, Riber-Hansen R, Jensen TO, Schmidt H, Steiniche T (2013). Proliferation indices of phosphohistone H3 and Ki67: strong prognostic markers in a consecutive cohort with stage I/II melanoma. Mod. Pathol..

[27] Alessi DR, Cuenda A, Cohen P, Dudley DT, Saltiel AR (1995). PD 098059 is a specific inhibitor of the activation of mitogen-activated protein kinase kinase in vitro and in vivo. J. Biol. Chem..

[28] Gopal YNV (2014). Inhibition of mTORC1/2 overcomes resistance to MAPK pathway inhibitors mediated by PGC1α and oxidative phosphorylation in melanoma. Cancer Res..

[29] Haq R (2013). Oncogenic BRAF regulates oxidative metabolism via PGC1α and MITF. Cancer Cell.

[30] Wang J (2018). A secondary mutation in BRAF confers resistance to RAF inhibition in a BRAF V600E-mutant brain tumor. Cancer Discov..

[31] Luebker SA, Koepsell SA (2019). Diverse mechanisms of BRAF inhibitor resistance in melanoma identified in clinical and preclinical studies. Front. Oncol..

[32] Johnson DB (2014). Combined BRAF (Dabrafenib) and MEK inhibition (Trametinib) in patients with BRAFV600-mutant melanoma experiencing progression with single-agent BRAF inhibitor. J. Clin. Oncol..

[33] Long GV (2014). Combined BRAF and MEK inhibition versus BRAF inhibition alone in melanoma. N. Engl. J. Med..

[34] Khavari TA, Rinn JL (2007). Ras/Erk MAPK signaling in epidermal homeostasis and neoplasia. Cell Cycle.

[35] Neuzillet C (2014). MEK in cancer and cancer therapy. Pharmacol. Therapeut.

[36] Flaherty KT (2012). Improved survival with MEK inhibition in BRAF-mutated melanoma. N. Engl. J. Med..

[37] Xiang M (1995). The Brn-3 family of POU-domain factors: primary structure, binding specificity, and expression in subsets of retinal ganglion cells and somatosensory neurons. J. Neurosci..

[38] Hartman ML, Czyz M (2015). Pro-survival role of MITF in melanoma. J. Invest. Dermatol..

[39] Wellbrock C, Arozarena I (2015). Microphthalmia‐associated transcription factor in melanoma development and MAP‐kinase pathway targeted therapy. Pigment Cell Melanoma Res..

[40] Haq R (2013). BCL2A1 is a lineage-specific antiapoptotic melanoma oncogene that confers resistance to BRAF inhibition. Proc. Natl Acad. Sci. USA.

[41] Zhang G (2016). Targeting mitochondrial biogenesis to overcome drug resistance to MAPK inhibitors. J. Clin. Invest.

[42] Smith MP (2019). A PAX3/BRN2 rheostat controls the dynamics of BRAF mediated MITF regulation in MITF(high)/AXL(low) melanoma. Pigment Cell Melanoma Res.

[43] Liu J, Fukunaga-Kalabis M, Li L, Herlyn M (2014). Developmental pathways activated in melanocytes and melanoma. Arch. Biochem. Biophys..

[44] Ganesh S (2018). β-Catenin mRNA silencing and MEK Inhibition display synergistic efficacy in preclinical tumor models. Mol. Cancer Ther..

[45] Sinnberg T (2016). A nexus consisting of beta-Catenin and Stat3 attenuates BRAF inhibitor efficacy and mediates acquired resistance to Vemurafenib. Ebiomedicine.

