# Supplementary material for: POU4F1 promotes the resistance of melanoma to BRAF inhibitors through MEK/ERK pathway activation and MITF up-regulation
Source: Cell Death Dis. 2020 Jun 12;11(6):451. doi: 10.1038/s41419-020-2662-2 (PMC7293281; doi:10.1038/s41419-020-2662-2)
Supplement: Supplementary file 1 — supplemental figure legend [file 41419_2020_2662_MOESM1_ESM.doc]

**Supplementary Figure Legend**

**Fig. S1 The establishment of acquired resistant melanoma cell models.** **A.** Viable cell titer was determined in parental and resistant cells under the treatment of Vemurafenib in concentration gradient, N=3. **B.** The expressions of ERK and p-ERK were analyzed by immunoblot in parental and resistant melanoma cells under the treatment of Vemurafenib in concentration gradient. Data are presented as the mean ± SEM. P: parental cells. VR: Vemurafenib-resistant cells. Vem: Vemurafenib.
