# Supplementary figures and images for: POU4F1 promotes the resistance of melanoma to BRAF inhibitors through MEK/ERK pathway activation and MITF up-regulation
Source: Cell Death Dis. 2020 Jun 12;11(6):451. doi: 10.1038/s41419-020-2662-2 (PMC7293281; doi:10.1038/s41419-020-2662-2)

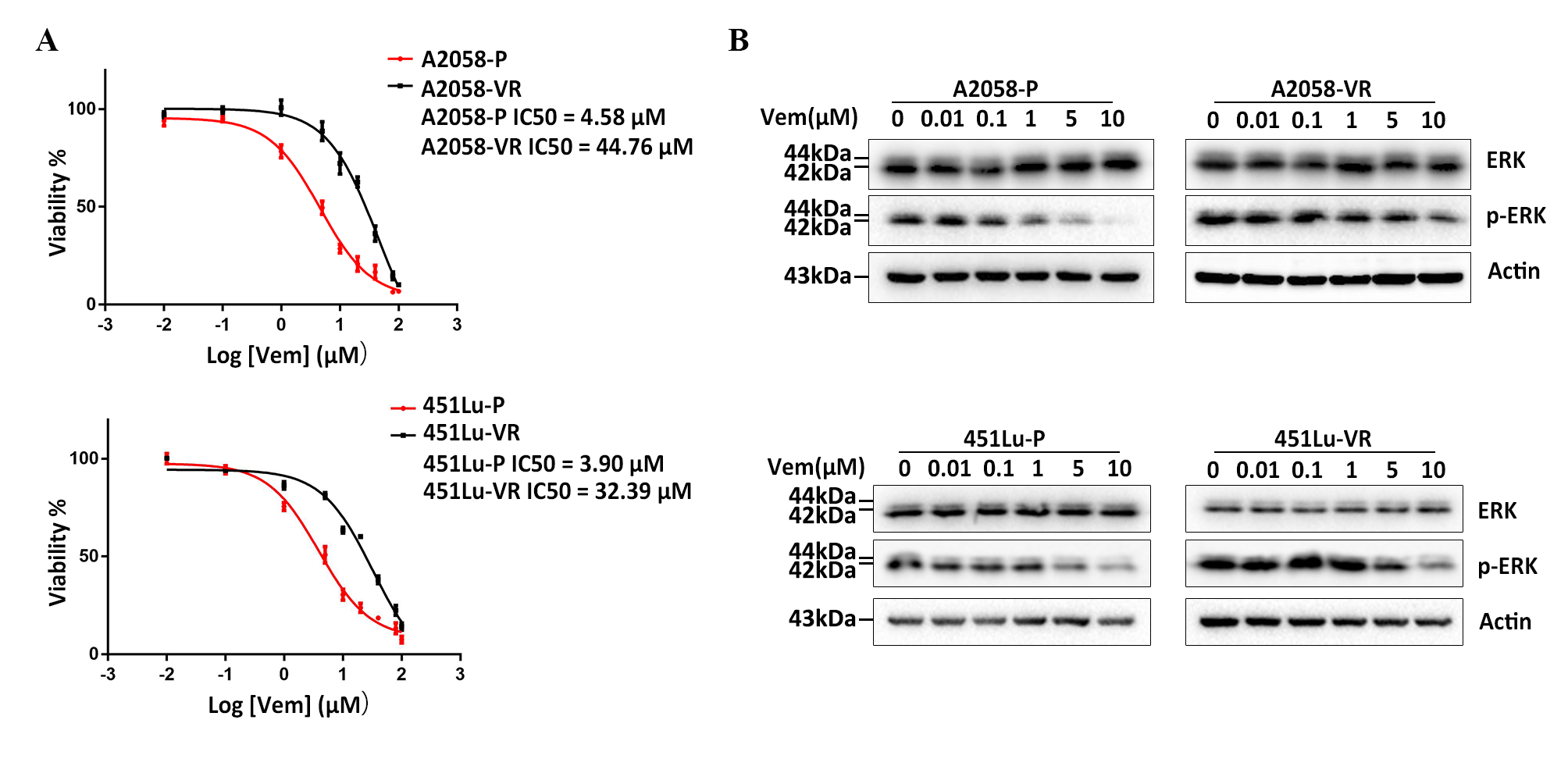

Supplement: Supplementary file 2 — supplemental figure S1 [file 41419_2020_2662_MOESM2_ESM.tif]
